# 162. The Genotypic Antibiogram: Using Results of Gram-Negative Antimicrobial Resistance Genes Identified via Rapid Blood Culture Identification Tests to Optimize Treatment of *Enterobacterales* Bloodstream Infections

**DOI:** 10.1093/ofid/ofad500.235

**Published:** 2023-11-27

**Authors:** Shawnalyn Sunagawa, Jeremy E Tigh, Ashlyn Okada, Scott J Bergman, Trevor C Van Schooneveld, Yolande Chan, Paul D Fey, Jonathan H Ryder

**Affiliations:** Nebraska Medicine, Omaha, Nebraska; Nebraska Medicine, Omaha, Nebraska; Nebraska Medicine, Omaha, Nebraska; Nebraska Medicine, Omaha, Nebraska; University of Nebraska Medical Center, Omaha, NE; University of Nebraska Medical Center, Omaha, NE; Univeristy of Nebraska Medical Center, Omaha, Nebraska; University of Nebraska Medical Center, Omaha, NE

## Abstract

**Background:**

With the increasing incidence of infections from *Enterobacterales* resistant to commonly used antibiotics, rapid diagnostic tests, such as BioFire’s Blood Culture Identification 2 (BCID2) multiplex PCR panel, can be utilized to detect potential antimicrobial resistance more rapidly and help direct more appropriate empiric therapy. We describe the epidemiology of *Enterobacterales* bloodstream infection (BSI), associated antimicrobial resistance genes using BCID2, and subsequent susceptibility patterns at an academic medical center to develop a genotypic antibiogram.

**Methods:**

We reviewed all positive BCID2 results at Nebraska Medical Center from 8/1/2021–11/1/2022. Only monomicrobial *Enterobacterales* BSI were included. Patient demographics, antimicrobial resistance markers, BCID2, culture, and susceptibility results were assessed. Sensitivity, specificity, positive predictive value (PPV) and negative predictive value (NPV) of bla_CTX-M_ marker were compared to ceftriaxone susceptibility patterns.

**Results:**

We reviewed 456 unique *Enterobacterales* isolates, of which 236 were from male patients (52%), 189 (41%) immunocompromised patients, and 342 (75%) community onset. The most common species identified were *Escherichia coli* (55%) and *Klebsiella pneumoniae* (17%). bla_CTX-M_ was detected in 49 (11%) isolates, with 41 (84%) *E. coli* and 6 (12%) *K. pneumoniae*. bla_KPC_ was detected in 2 isolates (1 *K. oxytoca* and 1 *K. variicola*), but no other carbapenemase genes were detected. bla_CTX-M_ sensitivity and specificity for detection of ceftriaxone resistance was 83% and 100%, respectively. bla_CTX-M_ PPV for ceftriaxone resistance was 100%, while the NPV of absent bla_CTX-M_ for ceftriaxone susceptibility was 97%.

**Conclusion:**

Locally, the majority of ceftriaxone-resistant *E. coli* and *Klebsiella* BSIs harbor bla_CTX-M_, while carbapenemases are rare. Utilization of rapid diagnostics can optimize antimicrobial therapy for *Enterobacterales* by creation of a genotypic antibiogram.

**Disclosures:**

**Scott J. Bergman, PharmD**, bioMerieux, Inc.: Honoraria **Trevor C. Van Schooneveld, MD, FSHEA, FACP**, AN2 Therapeutics: Grant/Research Support|Biomeriuex: Advisor/Consultant|Biomeriuex: Grant/Research Support|Insmed: Grant/Research Support|Thermo-Fischer: Honoraria

